# Case of Aneurism of the Arteria Innominata Treated by Pressure on the Distal Side

**Published:** 1858-10

**Authors:** 


### ECLECTIC DEPARTMENT,

### AND SPIRIT OF THE MEDICAL PERIODICAL PRESS.

---

*Case of Aneurism of the Arteria Innominata treated by Pressure on the Distal Side.* By Mr. EDWARDS, Demonstrator of Anatomy in the University of Edinburgh.

The mode of treatment which was employed successfully in the following case, does not appear to have been tried on any previous occasion:

CASE. In September, 1856, Mrs. L., æt. 50, was recommended to my care by Professor Simpson. She was a sallow-complexioned woman, with hanging, flabby cheeks; her lips, which were always apart, were livid and drawn down at the angles, and she breathed rapidly. Her countenance presented the peculiar anxious expression of one suffering from a fatal disease. On examining her neck, I found on the right side, above the sterno-clavicular articulation, a tumor, the size of an apple, situated between the sterno-mastoid muscle and the middle line of the neck, which pulsated violently, was soft and compressible, giving to the fingers much the same feeling as a vulcanized India-rubber ball, which, though easily compressible, expands again immediately the pressure is withdrawn; and with the expansion of this tumor, fluid seemed rapidly to fill the interior, and to be separated but by a thin partition from the fingers. Another pulsating tumor rose in front of the trachea. They were, though apparently distinct, evidently bulgings out of the same aneurism, as pressure on the one was followed by increase in size of the other. The patient had remarked these tumors about two months before I saw her, and they were, according to her account, increasing rapidly. Any pressure upon them was attended with pain and an increase of cough. She suffered from constant dyspnœa, had entirely given up her ordinary household occupations, and had frequent fainting fits; she rarely ventured even at night to lie down in bed, as, after falling asleep, the laryngeal spasm became so violent that an attendant had to be on the watch ready to administer restoratives. The ordinary internal remedies were tried but without much benefit.

As I considered that the aneurism was one of the innominate artery, I thought of placing ligatures on the vessels, according to Wardrop's method; but Dr. Laycock, who at my request examined her chest with the stethoscope, considered that the arch of the aorta was also aneurismal, so I gave up the idea of a cutting operation. But it struck me that Mr. Wardrop's principles could be applied to compression, and Mr. Young, of Prince's Street, constructed for me an instrument which I shall endeavor to describe.

A broad leathern belt to go round the chest, and fasten in front with three straps and buckles. On its left posterior and right anterior upper margins are brass buttons. In the middle of its posterior aspect is an iron plate perforated with several holes to admit screws, which attach to it an upright steel rod about eighteen inches long. This rod supports an arc of steel, which is attached to it by a screw allowing a certain amount of motion. In front, this arc is perforated by another screw, about an inch long, with a small cross handle; this projects backwards, and bears a conical piece of cork covered with wash leather. When the instrument is applied, the upright is at the back and right side of the neck, which rests in the arc, and, by shifting the lower end of the upright, pressure with the cork can be regulated and efficiently maintained upon the common carotid artery, the conical shape of the cork enabling one to confine the pressure to the artery. A strap is carried over the right shoulder from the bottons behind to those in front; on it slides another cone of cork, which can be adjusted over the subclavian. It will be seen that in principle this instrument resembles Bourgery's tourniquet for subclavian pressure.

Mrs. L. had worn this instrument for several hours for two days, when I was alarmed by observing that the tumor had visibly increased in size. Its walls felt thinner, and the contents were distinctly fluid. The *bruit de soufflet* was very loud, and the pulsation violent. The treatment, however, was continued, and the pads adjusted so as to stop all pulsation in the branches of the external carotid and in the right wrist. On the fourth day the tumor, though larger than when the instrument was first applied, was much harder and less compressible. The tracheal portion still, however, pulsated violently. Every morning, for the first two weeks, I adjusted the apparatus, at the same time manipulating the tumor rather roughly, with the view of breaking up the fibrin in the sac. But she soon learned how to apply it for herself, and, finding decided benefit from it, bore the treatment cheerfully. She said it was irksome, but never complained of pain.

After the first week the laryngeal symptoms entirely disappeared, and did not return, and she had no more fainting fits; but she complained of some impediment in swallowing, as if some hard body stopped the food in its passage down the gullet. I now feared that the apparent improvement was deceptive, and that the tumor was increasing towards the œsophagus; but as this symptom disappeared with the gradual decrease in the bulk of that part of the aneurism which we could judge of by external examination, I now conclude that it arose from the solidification of the contents of the sac in close apposition to the gullet.

By the end of three months she was well enough to lay aside the instrument and resume her former household duties. She repeatedly walked a distance of three miles and back to my house, and passed tranquil nights. The external part of the tumor was then, and is now, the size of a nut, and hard; the tracheal portion has entirely disappeared; the aortic aneurism seems to have made but little progress, and, with the exception of attacks of neuralgia in the face and head, and a chronic cough which troubles her every winter, she has been in good health since. She gave up the instrument more than ten months ago. Before she began to use it, a surgeon of great experience told me he expected the external tumor would burst in a day or two, and Dr. Laycock was of the same opinion. Of course, I cannot hope to avert the fatal termination we must always expect in thoracic aneurism; but I am convinced, and so is the patient, that the compression of the vessels beyond the aneurism was attended with marked benefit, and was the direct cause of its hardening and subsequent rapid decrease in its size.—*Ranking's Half-Yearly Abstract of the Med. Sciences.*

